# Efficacy of DaXianXiong Decoction in Preventing the Progression of Acute Pancreatitis Severity: Protocol for a Randomized Controlled Trial

**DOI:** 10.2196/67392

**Published:** 2025-04-29

**Authors:** Dongsheng Ren, Judan Tan, Yuling Zhou, Zhenchun Luo

**Affiliations:** 1 Department of Emergency and Intensive Critical Unit Chongqing Hospital of Traditional Chinese Medicine Chongqing China

**Keywords:** acute pancreatitis, traditional Chinese medicine, protocol, severe acute pancreatitis, prevent

## Abstract

**Background:**

Low- and middle-income countries are facing an increase in the incidence of acute pancreatitis (AP)—characterized by rapid onset, fast progression, high rate of severity, and high mortality. Progression of AP into severe AP (SAP) results in a series of complications such as organ dysfunction, local abscesses, pseudocysts, and necrosis. Although the treatment of AP is primarily supportive, including fluid resuscitation and organ support, there is still a lack of consensus on the optimal management regimen for fluid therapy, and strategies to promote gastrointestinal recovery remain limited. As no effective intervention measure has yet been developed, supportive therapy remains the primary approach for the early treatment of AP. DaXianXiong decoction is a widely used traditional Chinese medicine formulation; however, limited research has been conducted on its clinical efficacy.

**Objective:**

This study aims to evaluate the efficacy and safety of DaXianXiong decoction in preventing AP from progressing to SAP, assessing its impact on SAP incidence, clinical severity scores, inflammation markers, and gastrointestinal function, and providing evidence for AP management.

**Methods:**

This study is a randomized, double-blind, placebo-controlled, single-center clinical trial. The primary outcomes will include the incidence of SAP, modified computed tomography severity index score, APACHE II (Acute Physiology and Chronic Health Evaluation II) score, modified Marshall score, and levels of the inflammation factor. The secondary outcomes will include the effect of the gastrointestinal dysfunction treatment. Evaluations will be conducted at baseline; 24 hours after the intervention; and on days 3, 7, and 28 after the intervention in both groups. A total of 60 eligible patients will be randomly allocated in a 1:1 ratio to the intervention group and the control group. Both groups will receive standard Western medical treatment for pancreatitis. The intervention group will additionally receive DaXianXiong decoction, while the control group will receive a placebo similar to the decoction.

**Results:**

This study has been funded by the Performance Incentive Project of Scientific Research Institutions in Chongqing. The trial was registered in April 2024, and data analysis is expected to be completed by April 2025. The study results will be presented at both national and international conferences and published in peer-reviewed journals.

**Conclusions:**

This trial will help us assess the effectiveness and safety of DaXianXiong decoction in patients with AP and provide clinical evidence on the efficacy and safety of DaXianXiong decoction in preventing the progression of AP to SAP. By evaluating its impact, the findings will contribute to the understanding of DaXianXiong decoction as an adjunct therapy in AP management and may offer a novel complementary treatment strategy for AP, potentially improving patient outcomes and reducing complications.

**Trial Registration:**

Chinese Clinical Trial Registry ChiCTR2300076885; https://www.chictr.org.cn/showproj.html?proj=207084

**International Registered Report Identifier (IRRID):**

DERR1-10.2196/67392

## Introduction

Acute pancreatitis (AP) is a common yet potentially fatal inflammatory disorder of the pancreas, characterized by severe abdominal pain, multi-organ dysfunction, and pancreatic necrosis. The global incidence of AP is approximately 30-40 cases per 100,000 people, with over 2000 fatalities annually [1]. Severe AP (SAP), which involves persistent organ failure lasting ≥48 hours, is the most life-threatening form of AP, with a mortality rate of 20%-40% despite significant advancements in treatment [2-4]. Approximately 20% of patients with AP progress to SAP, which is associated with severe complications such as organ failure, infections, and increased health care costs [1,5].

The pathophysiology of AP is characterized by premature activation of trypsin within the pancreatic acinar cells, triggering pancreatic autodigestion and leading to a systemic inflammatory response. This response results in systemic inflammatory response syndrome, which can progress to multi-organ dysfunction syndrome [6,7]. Although the treatment of AP is mainly supportive, including fluid resuscitation and organ support, there remains a lack of consensus on the optimal management protocols for fluid therapy [8,9], and strategies to promote gastrointestinal recovery remain limited [10].

In addition to conventional Western treatments, Chinese herbal medicines have gained attention for their potential in managing AP. Chinese herbal medicines are known for their ability to target multiple biological pathways involved in inflammation, immune response, and organ protection, offering a broader approach compared to single-target Western therapies [11,12]. DaXianXiong decoction, a traditional herbal formula used in China for over 30 years, has been reported to improve gastrointestinal symptoms and modulate inflammation. However, its clinical efficacy in preventing the progression of AP to SAP has not been conclusively demonstrated due to the lack of robust randomized controlled trials [12-14].

In light of these challenges, there is an urgent need for high-quality studies to evaluate the efficacy and safety of DaXianXiong decoction in the treatment of AP. This study aims to assess the potential of DaXianXiong decoction in preventing the progression of AP to SAP, reducing inflammatory markers, improving gastrointestinal function, and assessing safety and complications.

## Methods

### Trial Design

This is a single-center, randomized, double-blind, placebo-controlled study. It adheres to the guidelines mentioned in the SPIRIT (Standard Protocol Items: Recommendations for Interventional Trials) statement (Multimedia Appendix 1).

**Figure 1:**
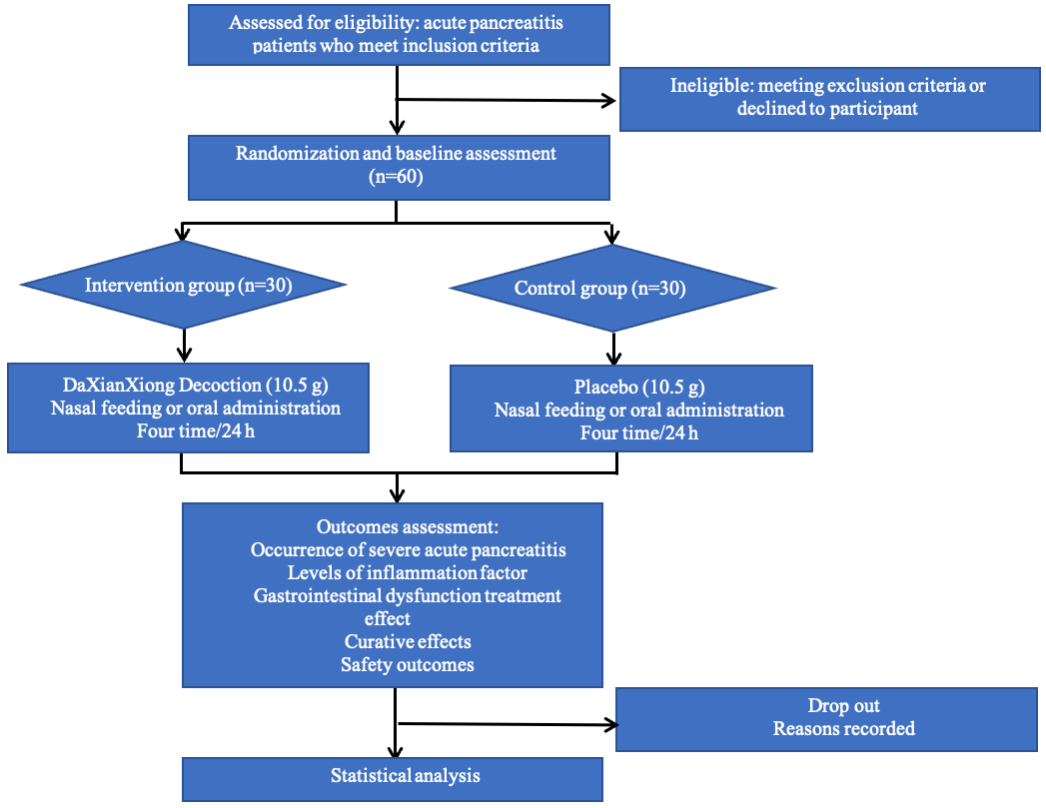
Workflow.

During the trial, all eligible patients will be randomly assigned to either the intervention group or the placebo group. Both groups will receive the standard treatment for AP. A detailed workflow depicting the study process is presented in Figure 1.

### Study Population

Patients will be enrolled from the intensive care unit in Chongqing Traditional Chinese Medicine Hospital, a grade III level A hospital in China.

### Recruitment of Patients

The study group will be recruiting and allocating the patients. Eligible patients who are willing to participate in the trial will be provided with an informed consent form with details of the study objectives, procedures, and potential risks (Multimedia Appendix 2).

### Patient and Public Involvement

Our research will not include any design, implementation, or reporting from patients or the public.

### Inclusion Criteria

We will select patients based on the following inclusion criteria:

- Presence of AP as confirmed using the revised Atlanta classification [15]
- Aged 18-75 years
- Time of illness onset under 24 hours

### Exclusion Criteria

We will use the following exclusion criteria:

- Patients with severe diseases affecting vital organs, such as liver dysfunction, heart failure, and malignant tumors
- Patients participating in another study within 3 months of enrollment
- Those with known allergies to any of the prescribed medicines
- Pregnant or lactating women

### Withdrawal Cases

Patients can withdraw from the study at any point without any repercussions. If a patient discontinues the intervention or decides to participate in another study, they will no longer be included in the present study. However, even when a patient withdraws from the study, the data collected up to that point will be retained and included in the analysis, unless specifically requested otherwise. The withdrawal and noncompliance of patients will also be documented. Incomplete data will be addressed based on the withdrawal rate. If the rate is <5%-10%, we will perform data cleaning. If the withdrawal rate exceeds 10%, the missing data will be managed through imputation, intention-to-treat analysis, and sensitivity analysis.

### Management of Dropout Cases

All patients who provide informed consent and meet the eligibility criteria for enrollment in this randomized controlled trial, irrespective of the timing or reasons for their withdrawal later, will be categorized as dropouts if they are lost to follow-up. However, patients who discontinued the treatment regimen because of complete symptom remission will not be considered dropouts. The investigators will comprehensively document the reasons for each dropout in the case report form. Real-world circumstances, including adverse events (AEs), will be accounted for during the statistical analysis. In addition, we will carefully record and preserve the data about all enrolled patients who will receive the assigned investigational medications, regardless of their withdrawal status, for observation purposes and to be used as a data archive and for the intention-to-treat analysis.

### Interventions

**Figure 2:**
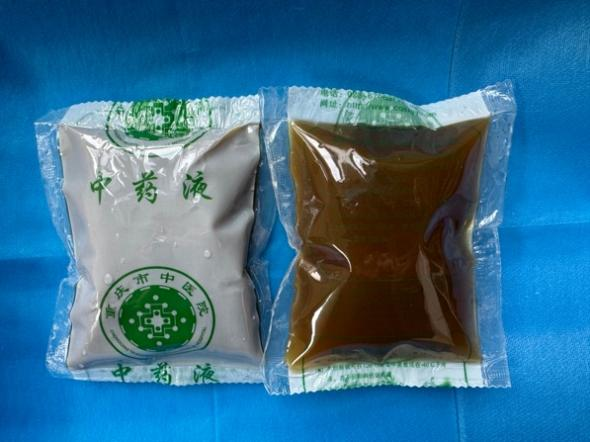
Components of DaXianXiong decoction.

All patients will be provided with detailed information about the study. Once informed consent is obtained and baseline assessments are completed, eligible patients will be randomly allocated to the intervention or placebo group. Notably, the investigational drug in this study was Chinese medicine, so patients were required not to take other Chinese medicine or Chinese patent medicine throughout the study. Both groups will receive standard treatments. In addition to the standard treatment, the intervention group will receive DaXianXiong decoction (Figure 2). The constituent botanical drugs of the DaXianXiong decoction are listed in Table 1. DaXianXiong decoction includes three herbs, namely, 30 g of Rhei Radix et Rhizoma (Shengdahuang), 30 g of Natrii Sulfas (Mangxiao), and 3 g of Kansui Radix (Gansui). The dosage of each herb was determined based on the guidelines outlined in *The Chinese Pharmacopoeia*, 2020 edition [16].

**Table 1:** Components of DaXianXiong decoction.

Drug name	Part this formula uses	Location of manufacture	Dose (g)	Use	Medicine form, dosage, frequency	Registration status (yes/no)	Quality control report (yes/no)	Storage condition
Da Huang (Rhei Radix et Rhizoma)	Roots	Sichuan Neautus Co, Ltd, Sichuan, China	7.5	Nasal feeding or oral administration	Powder, dissolved in 50 mL of warm water, every 6 h, lasts 48 h	Yes	Yes	Powder: stored in the pharmacy department; 18-20 °C and 45%-75% humidity Decoction: kept in the ward; 26-28 °C and 50%-60% humidity
Mang Xiao (Natrii Sulfas)	Mineral	Sichuan Neautus Co, Ltd, Sichuan, China	7.5	Nasal feeding or oral administration	Powder, dissolved in 50 mL of warm water, every 6 h, lasts 48 h	Yes	Yes	Powder: stored in the pharmacy department; 18-20 °C and 45%-75% humidity Decoction: kept in the ward; 26-28 °C and 50%-60% humidity
Gan Sui (Kansui Radix)	Roots	Sichuan Neautus Co, Ltd, Sichuan, China	0.5	Nasal feeding or oral administration	Powder, dissolved in 50 mL of warm water, every 6 h, lasts 48 h	Yes	Yes	Powder: stored in the pharmacy department; 18-20 °C and 45%-75% humidity Decoction: kept in the ward; 26-28 °C and 50%-60% humidity

The Department of Pharmacy of the Chongqing Traditional Chinese Medicine Hospital will perform the following processing steps on DaXianXiong decoction: water extraction, separation, concentration, drying, and granulation. Each sachet will contain 10.5 g of powdered decoction. The production and processing procedures will be conducted as per the guidelines specified in *The Chinese Pharmacopoeia*. The powder will be processed and filled in standardized clean production workshops. The treatment method will involve nasal feeding or oral administration of DaXianXiong decoction four times a day, once every 6 hours, and dissolved in 50 mL of warm boiled water. The course of the treatment will be 48 hours.

We will design placebos similar to the intervention drugs in terms of shape, color, smell, and solubility so that patients have a similar sensory experience. Placebos have no efficacy or side effects. They will also be produced by the Department of Pharmacy of the Chongqing Traditional Chinese Medicine Hospital.

In the event of a serious AE or allergic reaction caused by the research medication, the principal investigator will open the emergency envelope and discontinue the trial for that patient. Details of the event and unblinding will be recorded in the case report form, and the patient will receive appropriate treatment. The relevant departments and ethics committees will be promptly notified within 24 hours.

### Randomization

Randomization will be conducted by an independent statistician using SPSS Statistics 26.0 (IBM Corp) software. A predetermined random number table will be generated, and 60 patients will be randomly assigned to either the intervention group or the standard-of-care group in a 1:1 ratio. To ensure accuracy and reliability, a random distribution table will be created in triplicate, with copies provided to the project leader, pharmacist, and statistician. The statistician, who remains blind to the trial and is not involved in patient recruitment, will generate the allocation sequence and securely store the master randomization list. Randomization will be implemented using a sealed envelope method to maintain allocation concealment. Each patient will receive a unique study identification number upon enrollment. The randomization table will be used to sequentially assign these numbers to either the intervention or control group. Each assignment will be enclosed in an opaque, sequentially numbered, and sealed envelope. After obtaining informed consent, the recruiter will open the envelope corresponding to the patient’s study ID and determine the group assignment.

### Blinding

This study will be conducted as a double-blind trial to ensure that both patients and researchers remain unaware of group assignments. Each patient will be provided with a package containing medication numbers and labels. Random numbers will be securely sealed in double opaque envelopes and managed by a specialist (ZL). Each patient will be provided with their designated emergency letter, which will be carefully kept in secure storage until the study is completed. Discontinuation of the intervention and unblinding will only occur under specific circumstances: the patient experiences a drug-related serious AE, the patient fails to comply with the protocol regarding the administration of the investigational drug, or the investigator determines that continuing the trial may pose potential harm to the patient.

### Outcome Measures

The outcome measures will be determined at various time points throughout the study, including baseline (T0, before treatment), 24 hours after the final treatment (T1, end point 1), 3 days after the final treatment (T2, end point 2), 7 days after the final treatment (T3, end point 3), and 28 days after the final treatment (T4, final end point). The data collection and assessment schedule are listed in Table 2.

**Table 2:** The data collection and assessment schedule.

Assessments		Study period				Follow-up		
		Enrollment and allocation (T0)	Intervention (48 hours)	Primary end point (T1)	T2		T3	T4
**Enrollment**								
	Eligibility screen	✓						
	Informed consent	✓						
	Medical history	✓						
	Allocation	✓						
	Randomization	✓						
**Intervention**								
	Intervention group		✓					
	Placebo group		✓					
**Assessment**s								
	Incidence of SAP^a^			✓	✓		✓	✓
	Modified Marshall score	✓		✓	✓		✓	✓
	Modified CTSI^b^	✓		✓	✓		✓	✓
	APACHE II^c^ score	✓		✓	✓		✓	✓
	C-reactive protein	✓		✓	✓		✓	✓
	IL^d^-1	✓		✓	✓		✓	✓
	IL-4	✓		✓	✓		✓	✓
	IL-6	✓		✓	✓		✓	✓
	IL-8	✓		✓	✓		✓	✓
	IL-10	✓		✓	✓		✓	✓
	TNF^e^-α	✓		✓	✓		✓	✓
	Safety outcomes	✓		✓				
	Hospitalization							✓
	Survival							✓

^a^SAP: severe acute pancreatitis.

^b^CTSI: computed tomography severity index.

^c^APACHE II: Acute Physiology and Chronic Health Evaluation II.

^d^IL: interleukin.

^e^TNF: tumor necrosis factor.

### Primary Outcomes

The primary outcome of this study is the incidence of SAP, which will be calculated using the following formula:

SAP incidence (%) = (number of SAP cases/total number of patients) × 100%

### Secondary Outcomes

#### Modified Computed Tomography Severity Index

According to the analysis of the area under the curve, the modified computed tomography severity index demonstrates the highest accuracy in predicting SAP. It is a simpler and more accurate scoring tool, showing a stronger statistical correlation with factors such as length of hospital stay, development of infection, organ failure, and mortality [17,18]. It will be determined using the following formula:

Computed tomography severity index = (original score – current score)/original score × 100%

#### APACHE II Score

The APACHE II (Acute Physiology and Chronic Health Evaluation II) scoring system has high sensitivity and exhibits a negative predictive value in predicting pancreatic necrosis and organ failure. This makes it an ideal prognostic tool for assessing the severity of AP and in guiding clinical decision-making, particularly in resource-limited countries [19]. It is determined using the following formula:

APACHE II = (original score – current score)/original score × 100%

#### Modified Marshall Score

Organ failure plays a significant role in the severity of AP. The revised Atlanta classification recommends the use of the modified Marshall scoring system as the primary tool for assessing organ failure. The modified Marshall scoring system encompasses scoring systems for respiratory, cardiovascular, and renal systems to evaluate organ dysfunction in AP [6].

#### Levels of Inflammation Factor

The level of an inflammation factor is a surrogate marker for important clinical end point variables in AP. Multiple studies have shown that C-reactive protein is a good surrogate marker for SAP [20,21]. Additionally, interleukin (IL)–6 is superior in predicting SAP compared to other commonly used markers such as erythrocyte sedimentation rate. The level of an inflammation factor can be determined using the following formula [22]:

Inflammation factor = (original figure – current figure)/original figure × 100%

We will also use IL-1, IL-4, IL-8, and IL-10 to investigate the potential targets of DaXianXiong decoction. In pancreatitis, cytokines such as tumor necrosis factor–α secreted by acinar cells and damage-associated molecular patterns play a major role in activating immune cells such as neutrophils and macrophages. This process leads to increased pancreatic damage, systemic inflammation, and the development of SAP [8,23].

#### Gastrointestinal Dysfunction Treatment Effect

A comprehensive assessment of the gastrointestinal function is essential for the early detection of SAP. This assessment involves evaluating symptoms such as abdominal pain, distention, hematemesis, hematochezia, number of independent defecations, and digestive function. Signs such as tenderness, rebound tenderness, muscle tension, and the number of bowel sounds are also considered. Additionally, scores such as the gastrointestinal function score used in multi-organ dysfunction syndrome diagnostic criteria can provide a standardized measurement of gastrointestinal function.

#### Other Measures

Given the constraints of ethics and experimental techniques, certain indicators are valuable for assessing the improvements and prognosis of patients with AP. These include the time to bowel sound recovery, time to first defecation, and the gastrointestinal function score. These measures provide predictive information regarding gastrointestinal function and can be useful in evaluating the prognosis of patients with AP. We will also record hospitalization costs and duration.

### Safety Outcomes

The safety outcomes of the investigational medication were assessed at T0, T1, T2, T3, and T4 by considering the following:

- Measurement of vital signs: heart rate, blood pressure, temperature, and respiration
- Laboratory tests: blood routine, stool routine, and urine routine
- Liver function tests: alanine aminotransferase, aspartate aminotransferase, and bilirubin
- Renal function tests: urea nitrogen and creatinine
- Electrocardiogram

### Adverse Events

All AEs will be carefully documented, including the start and end dates, severity, relationship to the study drug, and patients’ continued involvement in the study. Any serious AE will be promptly reported to the research ethics committee within 24 hours. If an AE occurs, the patient will be advised to discontinue the use of the decoction, and the researchers will evaluate whether it is related to the formulation. Emergency safety measures will be implemented, if necessary, to ensure the well-being of patients. Persistence of AEs will be monitored until their resolution. Please note that AEs are not the complications clearly related to AP, such as acute respiratory distress syndrome, abdominal compartment syndrome, and acute kidney injury.

### Sample Size

We will use preset sample sizes (*n*) determined using the following formula:

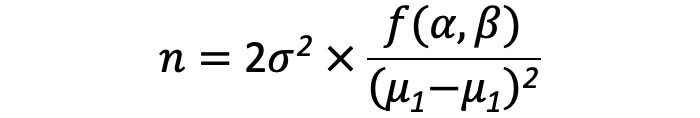



Where *μ*_1_ and *μ_2_* are the means of the intervention and placebo groups, respectively, and *σ* is the SD. The study aims to maintain the type I error probability (*α*) to <5% and the type II error probability (*β*) to <10%, with the *α* at .05 and the *β* at .1. To determine the sample size, we referred to literature reports in Chinese [24] on the use of DaXianXiong decoction for the treatment of AP. We used the time blood amylase took to return to normal levels to determine the clinical efficacy of the decoction. We fixed the total sample size for the study at 60. We allocated 30 patients to the intervention group and the remaining 30 to the placebo group.

### Data and Sample Collection

To minimize selection bias, three study members will receive comprehensive training on the criteria and methods used for case collection. Detailed records of patient medication will be collected, and any interfering or contaminating factors will be excluded from the statistical analysis.

### Data Management and Monitoring

An independent data and safety monitoring board will be established to monitor the conduct and safety of the study. Two of our research team members will be responsible for updating our clinical trial–related information on the Chinese Clinical Trial Registry website and for making sure that no data are missed. However, if this becomes inevitable, we will use various processing methods to handle the issue. We will also perform a sensitivity analysis of the test results simultaneously.

### Adherence to Study Interventions

Throughout the study, patients will be given clear oral and written instructions to ensure proper adherence to the intervention. We will closely monitor their progress and provide personalized guidance when necessary. Any unused medication will be collected, documented, and returned as part of the study protocol.

### Data Analysis

Data analyses will be performed using SPSS 24.0 software. Continuous variables with a normal distribution will be analyzed using Student *t* test, while nonnormally distributed variables will be analyzed using the Wilcoxon rank sum test. Categorical variables will be analyzed using Pearson *χ*^2^ test. Continuous variables will be presented as mean (SD), while categorical variables will be presented as percentages. Values with *P* values ≤.05 will be considered statistically significant, and all tests will be 2-sided.

### Frequency and Plans for Auditing Trial Conduct

The trial conduct will be audited at specified frequencies by an independent team that does not include the investigators and sponsor. The details of the auditing frequency and plans are provided in Table 2.

### Ethical Considerations

This study protocol involving human patients was reviewed and approved by the Chongqing Traditional Chinese Medicine Hospital on September 6, 2022 (2023-KY-52).

Patients will be provided with a consent form with a clear outline of the use of their data, allowing them to choose whether to continue participating or withdraw from the trial. Biological specimens collected for storage will be handled according to ethical guidelines, and they will be destroyed after their intended use. Patient privacy will be strictly maintained. Physical documents related to the study will be secured in a locked office.

### Modification of the Protocol

Any modifications to the program will require the consent of both the project leader and the supervisor. After obtaining approval from the ethics committee, the sponsor and all members of the research team will be notified of the changes.

## Results

This study has been funded by the Performance Incentive Project of Scientific Research Institutions in Chongqing. The trial was registered in April 2024, and data analysis is expected to be completed by April 2025. The recruitment for the trial commenced in December 2023. The data has been collected and is being analyzed. The study results will be presented at both national and international conferences and published in peer-reviewed journals.

## Discussion

### Expected Findings

This study aims to evaluate the efficacy and safety of DaXianXiong decoction in preventing the progression of AP to SAP by conducting a randomized, double-blind, placebo-controlled, single-center trial. The treatment of SAP continues to be a challenge, and preventing the progression of AP to SAP has been a hot spot of research in critical care medicine and gastrointestinal diseases. Inflammatory cascade-driven response is considered to be an important cause of progression to SAP in patients with AP combined with organ failure [25]. Inflammatory response to AP triggers visceral edema and gastrointestinal dysfunction, leading to increased abdominal pressure and possible abdominal septal compartment syndrome and organ failure [26]. Among the studies on blocking the progression of AP to SAP, Huang et al [27] observed that cyclooxygenase 2 (COX-2) inhibitors (parecoxib and celecoxib) reduced serum tumor necrosis factor–α and IL-6 levels in patients with AP, reducing the incidence of SAP by approximately half. He et al [28] observed that neostigmine reduced AP and abdominal pressures and facilitated defecation, thereby reducing the incidence of organ dysfunction in patients with AP. However, these drugs have a single therapeutic target and have potential risks, such as COX-2 inhibitors that may lead to gastrointestinal mucosal damage or even gastrointestinal bleeding, and neostigmine that may cause bradycardia and increase airway resistance.

DaXianXiong decoction has been commonly used in Asia for the treatment of AP for more than 30 years [29,30]. In our study, we used DaXianXiong decoction in addition to conventional treatment to observe its efficacy in preventing the progression of AP to SAP. The key advantage of this study compared with previous studies is that DaXianXiong decoction has the advantage of being multi-targeted to simultaneously act to improve gastrointestinal function and reduce the inflammatory response, which has the potential to block the progression of AP. In addition, this study may pave the way for larger multicenter trials to further investigate the potency and cost-effectiveness of complementary treatment strategies in larger populations.

### Limitations

The study has limitations because of the small sample size. Additionally, the mechanism path of DaXianXiong decoction remains unclear and requires further investigation. However, these limitations provide further opportunities for expanding the findings and gaining a deeper understanding of the treatment.

## Appendix

Multimedia Appendix 1 SPIRIT checklist.

Multimedia Appendix 2 Informed consent.

## Abbreviations

AE: adverse event
AP: acute pancreatitis
APACHE II: Acute Physiology and Chronic Health Evaluation II
COX-2: cyclooxygenase 2
IL: interleukin
SAP: severe acute pancreatitis
SPIRIT: Standard Protocol Items: Recommendations for Interventional Trials

## Data Availability

The datasets generated or analyzed during this study are available from the corresponding author upon reasonable request.
