# Supplementary material for: Efficacy of DaXianXiong Decoction in Preventing the Progression of Acute Pancreatitis Severity: Protocol for a Randomized Controlled Trial
Source: JMIR Res Protoc. 2025 Apr 29;14:e67392. doi: 10.2196/67392 (PMC12076030; doi:10.2196/67392)
Supplement: Multimedia Appendix 2 [file resprot_v14i1e67392_app2.doc]

附件：AF/FJ-04/03.0

**基于“截断”理论早期应用大陷胸汤改善急性胰腺炎重症化趋势的临床研究知情同意书**

尊敬的受试者/受试者法定代理人：

我们邀请您或您的代理人参加2021年重庆市科研机构绩效激励引导专项项目批准开展的《基于“截断”理论早期应用大陷胸汤改善急性胰腺炎重症化趋势的临床研究》课题研究。本研究将在重庆市中医院开展，估计将有108名受试者自愿参加。本研究已经得到重庆市中医院伦理委员会的审查和同意。

**为什么要开展本项研究?**

重症急性胰腺炎是指急性胰腺炎加重出现机体脏器功能不全，或出现局部脓肿、假性囊肿及坏死等一系列并发症的炎症性疾病。近年来，急性胰腺炎发病率逐年增加，具有起病急、进展快、重症化率高、病死率高的特点。

急性胰腺炎的发生是由复杂的、多因素参与的病理生理过程，而病程早期炎症因子级联释放以及胃肠动力障碍是导致急性胰腺炎重症化，出现全身炎症反应综合征(SIRS)、持续性器官功能衰竭(POF)以致死亡的重要因素，早期的治疗成败对病程中后期并发感染、局部囊肿等有重要的影响。目前, 急性胰腺炎早期治疗以液体复苏、促进胃肠动力恢复、早期营养支持等对症治疗为主。但液体复苏容易引起组织间隙水肿并发ARDS、腹高压，甚至是中后期局部并发症的独立风险因素。关于促进胃肠动力的恢复，尚无切实有效干预措施。针对重症急性胰腺炎，国内大部分临床机构早期仍以禁食、胃肠减压为主要手段，未能早期肠内营养治疗。因此，在病程早期进行干预，阻断炎症风暴，恢复胃肠动力，弥补常规治疗的不足，改善重症化趋势，进一步改善预后，至关重要。

“截断”理论是指在疾病发生以后，应早期干预，以防止疾病进展与传变，体现了中医药治未病，既病防变的特色优势。大陷胸汤出自《伤寒论》，大陷胸汤之主症与急性胰腺炎早期临床表现完全契合。大陷胸汤由生大黄、芒硝、醋甘遂组成，方中醋甘遂为君药，苦寒峻下，善攻逐经隧脉络之水饮；生大黄泻热通便，荡涤肠胃；芒硝可泻下通便，润燥软坚；三药合用，力专效宏，是泄热逐水、开结通便的峻剂。运用中医“取向比类”方法，机体免疫失调导致炎症因子及炎性介质的产生和表达，肠道功能障碍产生的大量内毒素，液体复苏导致肠壁、肺间质等组织间隙的水分，正是伏于肠胃、经隧、脉络之水饮热毒邪气，热毒水饮互结，胸腹气机闭塞，三焦不通，继而并发暴喘、闭证、隆闭、神昏、脉微而死等危候，如现代医学之ARDS、腹腔间隔室综合征、急性肾衰竭、休克等。热毒炽盛、水饮弥漫，水热互结，气机闭塞，须臾则变证丛生，且六腑以通为用，故宜尽早应用大陷胸汤峻下逐水、泄热解毒、开结通腑。

前期实验及临床研究已证实生大黄、芒硝、醋甘遂可通过改善患者免疫功能紊乱，抑制炎性因子的表达，减少炎症介质的产生和释放，缩短腹痛、腹胀及胃肠功能恢复时间，减少感染等并发症。

本研究拟在病程早期使用大陷胸汤治疗急性胰腺炎，用客观数据验证大陷胸汤可通过降低炎症反应、促进胃肠功能恢复降低急性胰腺炎重症化趋势，为中医药在重症急性胰腺炎的临床诊疗中提供科学的试验依据。

本研究受试人群的纳入标准是年龄在18-80岁之间，发病时间24小时以内的急性胰腺炎患者。本研究将在重庆市中医院中心ICU展开，预计有108余名受试者自愿参加。

**如果参加研究，需要做什么?**

如果同意您或您的代理人参加本项研究，对照组患者将给予西医基础治疗，治疗组在给予内科治疗的基础上辨证口服中药（大陷胸汤），给药时间为从经过评估患者可以进食或启动肠内营养后立即给予大陷胸汤治疗（大陷胸汤一剂由生大黄30克、芒硝30克、醋甘遂3克组成）。治疗方式为每天鼻饲或者口服一剂，分四次使用，每6小时一次，使用疗程为48小时。我们将评估重症发生率、改良Marshall评分、APACHEⅡ评分、MCTSI评分、Ranson评分、炎症因子水平（CRP、中性粒细胞比、白介素1、白介素6、白介素8、肿瘤坏死因子α）、腹痛、腹胀、恶心呕吐等中医症状积分，肠鸣音恢复时间，腹腔压力（膀胱压）下降率，肛门首次排气、排便时间、28天病死率，平均ICU住院时间。并临床观察和实验室检测结果等。

**哪些人不宜参加研究?**

排除标准，如果您或您的代理人为满足以下条件的患者，不属于本次研究范畴：（1）妊娠期、哺乳期，或已知有生育要求的患者；（2）对本研究方案中使用的药物有过敏的患者；（3）基础疾病合并有严重肝功能衰竭、肾功能衰竭、心血管系统、血液系统等器官功能不全或有进展性恶性肿瘤的患者；（4）经外科会诊后适合手术治疗者；（5）有严重精神、心理或神经疾患不能与配合研究。

**参加研究有哪些风险?**

所有治疗药物都有可能产生副作用，根据患者具体不良反应情况，采取对症支持治疗，如大陷胸汤可能引起过敏性反应、胃肠道刺激反应、肝损伤等，对试验期间出现的不良事件、不良反应，根据患者具体情况，采取对症支持治疗，并将其种类、程度、出现时间、持续时间、处理措施、处理经过等记录于CRF表，并且在综合考虑合并症、合并用药的基础上，评价其与试验药物的相关性，并由医师详细记录。若与试验药物相关，将由本课题组承担所有不良反应相关的治疗及检查费用。

**参加研究有哪些好处?**

参加本项研究，您或您的代理人的病情有可能获得改善，本项研究还有助于确定哪种治疗方法可以更安全有效地治疗与您或您的代理人患有相似病情的其他病人。

**参加研究需要支付有关费用吗?**

为了补偿您或您的代理人参加本研究可能给您带来的不便，本研究将免费为您或您的代理人提供研究期间所使用的药物（中药制剂）。治疗期间常规治疗药物及检查项目不在免费范围之内。如果您或您的代理人同时合并其他疾病所需的治疗和检查，以及因治疗无效而改用其他治疗的费用，将不在免费的范围之内。我们将通过定期随访观察药物治疗可能引起的副作用/不良反应，并采取措施加以防治，但是如果出现试验药物引起的不良反应，所需的治疗和检查费用将由课题组支付。

**个人信息是保密的吗?**

您或您的代理人的医疗记录将保存在医院，研究者、研究主管部门、伦理委员会将被允许查阅您的医疗记录。任何有关本项研究结果的公开报告将不会披露您或您的代理人的个人身份。我们将在法律允许的范围内，尽一切努力保护您或您代理人的个人医疗资料的隐私。

**我必须参加研究吗?**

参加本项研究是完全自愿的，您或您的代理人可以拒绝参加研究，或在研究过程中的任何时间退出本研究，这都不会影响医生对您或您代理人的治疗。如果您或您的代理人不参加本项研究，中途退出研究，还有很多可替代的治疗药物。如果您或您的代理人决定退出本研究，请与您或您代理人的医生联系，您或您的代理人可能被要求进行相关检查，这对保护您或您的代理人的健康是有利的。

**受试者声明:**我已经阅读了上述有关本研究的介绍，对参加本研究可能产生的风险和受益充分了解。我自愿参加本研究。

我**同意口**或**拒绝口**除本研究以外的其他研究利用我的医疗记录和病理检查标本。

受试者签名： 日期：

受试者的联系电话： 手机号:

法定代理人签名(如适用)： 日期：

法定代理人的联系电话: 手机号:

**研究者声明:**我确认已向受试者解释了本研究的详细情况，特别是参加本研究可能产的风险和受益。

研究者签名: 日期:

研究者的工作电话: 手机号:

医院伦理委员会办公室联系电话:023-67630637

地址：南桥寺院部行政楼三楼伦理办公室
